# Diversity of gastrointestinal parasites in sympatric mammals in Moukalaba-Doudou National Park, Gabon

**DOI:** 10.14202/vetworld.2021.3149-3155

**Published:** 2021-12-25

**Authors:** Serge-Ely Dibakou, Ulrich Maloueki, Barthélémy Ngoubangoye, Larson Boundenga, Stephan Ntie, Thierry-Audrey Tsoumbou, Cyr Moussadji, Rina Obame Zang, Dikenane Kombila, Didier Basset

**Affiliations:** 1 Centre de Primatologie, Centre Interdisciplinaire de Recherches Médicales de Franceville (CIRMF), BP 769 Franceville, Gabon; 2 Department of Biology, Faculty of Sciences, Kinshasa University, PO Box 190, Kinshasa XI; 3 Protectrice des Grands Singes de la Moukalaba (PROGRAM), PO Box 861, Libreville, Tchibanga, Gabon; 4 Groupe Evolution et Transmission Inter-espèces de Parasites (GETIP) du Département de Parasitologie, Centre Interdisciplinaire de Recherches Médicales de Franceville, BP 769 Franceville, Gabon; 5 Département de Biologie, Laboratoire de Biologie Moléculaire et Cellulaire, Université des Sciences et Techniques de Masuku, BP 941, Franceville, Gabon; 6 Parasitology Laboratory, CHU Montpellier, Montpellier, France

**Keywords:** conservation, coprology, gastrointestinal parasites, Moukalaba-Doudou National Park, wildlife mammals

## Abstract

**Background and Aim:**

Gastrointestinal parasites identified in the wild can negatively affect host fitness, lower performance, and growth. On the other side, sympatric mammals that share habitat and resources may also cross-transmit parasites, which are often zoonotic and can contribute to morbidity and mortality. This study aimed to characterize the diversity of gastrointestinal parasites circulating in mammalian hosts in Moukalaba-Doudou National Park.

**Materials and Methods:**

We screened a total of 25 fecal samples collected from nine wild mammalian species, namely, western gorilla (*Gorilla gorilla gorilla*), chimpanzee (*Pan troglodytes*), putty-nosed monkey (*Cercopithecus nictitans*), African forest elephant (*Loxodonta cyclotis*), African buffalo (*Syncerus caffer*), blue duiker (*Philantomba monticola*), bay duiker (*Cephalophus dorsalis*), and red river hog (*Potamochoerus porcus*) as well as people working as trackers (*Homo sapien*s) using direct microscopic observations following a sedimentation technique to concentrate the fecal material.

**Results:**

Of the total 25 fecal samples screened, 15 (60%) were positive for parasitic gastrointestinal infection. Based on the morphology of parasite eggs and cysts, we identified a rich diversity of nematodes, protozoans, trematodes, and cestodes, including unidentified strongyles (73%), *Oesophagostomum* spp. (53%), *Ancylostoma* spp. (27%), *Trichuris* spp. (13%), *Ascaris* spp. (13%), *Mammomonogamus* spp. (13%), *Strongyloides* spp. (47%), *Balantidium coli* (20%), *Entamoeba coli* (20%), *Endolimax nana* (6%), *Fasciola hepatica* (6%), *Paramphistomum* spp. (13%), and *Taenia* spp. (6%).

**Conclusion:**

All parasites were found at least once in one of the hosts, and most were potentially zoonotic and responsible for several diseases of public health concern. Because of the small sample size, our findings should not be considered conclusive. Nevertheless, they highlight the diversity of gastrointestinal parasites in this area.

## Introduction

Gastrointestinal parasites play an important role in mammalian health and survival because they impact host fitness. Thus, they are a major concern in the conservation of threatened species [1,2]. Intestinal parasites are among the most common infections worldwide, and they lead to morbidity and mortality as well as indirect ecologic pressure that influence community structure, trophic interactions, genetic variability, food web characteristics, and population decline [3-5]. The most common gastrointestinal parasites infecting mammalian species are protozoans, nematodes, and trematodes, which are widespread in most environments [1,6].

The occurrence and prevalence of gastrointestinal parasites in mammals vary according to the geographic location, season, and habitat type [7]. For instance, tropical regions with dense equatorial forests, moist conditions, and high humidity appear to favor a diverse range of parasite eggs [8,9]. In addition, a variety of infectious diseases originating from mammalian hosts, including bats, rodents, and non-human primates, are important threats to human health. Moreover, the close phylogenetic proximity between non-human primates and humans and the expansion of human activities (e.g., mining, bushmeat hunting, farming, and logging) into the areas previously uninhabited by people [10,11] increases the probability of the transmission of zoonotic pathogens to humans and *vice versa*. Thus, documenting the nature of the pathogens circulating in wild populations has become a priority. For example, Ebola hemorrhagic fever and anthrax outbreaks have decimated populations of African great apes [12,13] and led to a decline in several other taxa, such as amphibians, African carnivores, and African monkeys [14,15].

This study is a preliminary attempt to characterize the gastrointestinal parasites circulating in mammalian hosts in Moukalaba-Doudou National Park (MDNP), Southwest Gabon. This park is classified as a Category II protected area by the International Union for Conservation of Nature and was listed as a World Heritage site by the United Nations Educational, Scientific, and Cultural Organization in 2005 because of its exceptionally rich fauna and flora populations. Due to its species diversity and endemism, the presence of rare and endangered species, and a high density of western gorillas and the resultant ecotourism activity based on their habituation [16], MDNP is of great interest. However, despite the high level of species richness, little is known about the gastrointestinal parasites that infect mammals inhabiting the park. Although limited data are available for wild lowland gorillas [17-19], there is a clear need for research to assess the distribution and prevalence of gastrointestinal parasites in wild animals.

In this context, this study aimed to characterize the diversity of gastrointestinal parasites circulating in mammalian hosts in Moukalaba-Doudou National Park.

## Materials and Methods

### Ethical approval

The study was approved by the National Ethics Committee of Gabon and with the authorization of the Gabonese Ministries of Water and Forestry, Higher Education, Scientific Research and Innovation (NͦAR0031/09/MENESRESI/CENAREST/CG/CST/CSAR).

### Study period and site

**Figure-1:**
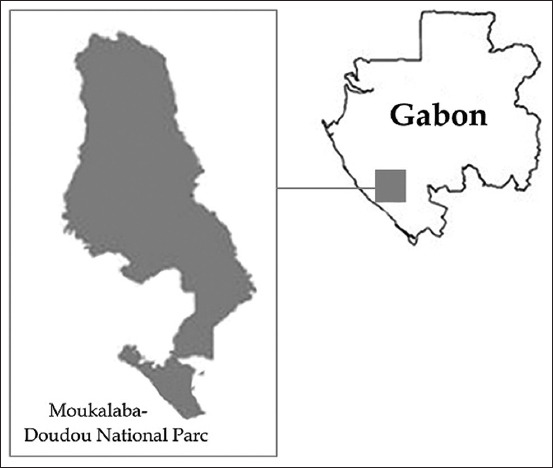
Moukalaba-Doudou National Park in Gabon [20].

We conducted this field study on September 23-24, 2019, during the monitoring of a habituated gorilla group in MDNP (Figure-1). Trained local trackers monitor this gorilla group as part of the Protectrice des Grands Singes de la Moukalaba (PROGRAM) project. This park covers 5028 km^2^ and consists of a mix of vegetation types, including primary forest, secondary forest, riverine forest, montane forest, and savanna [16]. Our research camp, Douguetsi (about 23.01km²), is located on the northeast side of the park, at the following coordinates 02°22’11.42”S, 10°33’44.53”E, approximately from 6 km from Doussala village along the right side of the Moukalaba River.

### Sampling

We opportunistically collected fresh fecal samples as soon as possible after defecation during routine reconnaissance patrols of the habituated gorilla group [20]. We collected a total of 25 fecal samples from nine species of wild mammals. Five species were non-human primates, including western gorilla (*Gorilla gorilla gorilla*), chimpanzee (*Pan troglodytes*), putty-nosed monkey (*Cercopithecus nictitans*), and African forest elephant (*Loxodonta cyclotis*), and four species were ungulates, including African buffalo (*Syncerus caffer*), blue duiker (*Philantomba monticola*), bay duiker (*Cephalophus dorsalis*), and red river hog (*Potamochoerus porcus*) (Table-1). We also collected stool samples from people working as trackers (*Homo sapien*s) after explaining the purpose of our study and obtaining their signed consent. Fresh samples were stored at ambient temperature at the PROGRAM base camp for a maximum of 3 days before being taken to the pathology laboratory at the Centre Interdisciplinaire de Recherches Médicales de Franceville (CIRMF) for parasitological examination.

**Table 1:** Prevalence of the gastrointestinal parasites observed in sampled mammals.

Host species	Samples collected	Positive	Parasites observed												

			Nematoda							Protozoa			Trematoda		Cestoda
			Unidentified	Oeso	Ancylo	Tri	Asc	Mamo	Strong	Bal	Ecoli	Enana	Facio	Parza	Taenia
Western gorillas	6	3	3	2	2	-	1	1	2	-	-	-	-	1	-
Chimpanzees	3	2	3	1	-	1	1	1	1	1	-	-	-	-	-
Putty-nosed monkey	1	1	-	-	-	-	-	-	-	1	1	-	-	-	1
African elephants	3	3	2	3	1	-	-	-	3	-	-	-	-	1	-
African buffalo	1	0	-	-	-	-	-	-	-	-	-	-	-	-	-
Blue duiker	3	2	1	-	-	-	-	-	-	-	-	-	-	-	-
Bay duiker	1	1	-	-	-	-	-	-	-	-	-	-	1	-	-
Red river hog	1	1	2	1	-	-	-	-	1	1	1	-	-	-	-
Local trackers	6	2	-	1	1	1	-	-	-	-	1	1	-	-	-
Total and percent prevalence	25	15 (60%)	11 (73%)	8 (53%)	4 (27%)	2 (13%)	2(13%)	2 (13%)	7(47%)	3 (20%)	3 (20%)	1 (6%)	1(6%)	2 (13%	1 (6%)

Unidentified=Strongylid egg, Oeso=*Oesophagostomum* spp., Ancylo=*Ancylostoma* spp., Tri=*Trichuris* spp., Asc=*Ascaris* spp., Mamo=*Mammomonogamus* spp., Strong=*Strongyloides* spp., Bal=*Balantidium coli,* Ecoli=*Entamoeba* *coli*, Enana=*Endolimax nana*, Facio=*Fasciola hepatica*, Para=*Paramphistomum* spp., and *Taenia* spp.

### Parasite identification

Direct microscopic observations were performed after using a sedimentation technique to concentrate the fecal material. Briefly, approximately 1 g of fecal material was weighed out and added to 6 mL of Bailenger solution (15 g sodium acetate, 3.60 mL acetic acid, and 1000 ml distilled water) in a Parasep filter fecal concentrator tube (manufactured by VWR International, France). Then, the solution was mixed using a tongue depressor and vortexed. Next, the homogenized solution was centrifuged at 1225 x g (1500 rpm) for 3 min, and 4 mL of the supernatant was gently decanted. Finally, 20 mL of the sediment was pipetted onto a glass slide and covered with a coverslip (22×32 mm). The slide was examined at 10×, 40×, and 100× for parasite identification [21]. Helminth eggs, protozoan trophozoites, and cystic stages were identified according to their morphologic characteristics (e.g., shape, size, color, and internal and external structure of eggs, cysts, or trophozoites) [22,23].

## Results

Of the total 25 fecal samples screened, 15 (60%) were positive for parasitic gastrointestinal infection (Table-1). Based on morphologic characteristics of the parasite eggs and cysts, we identified a rich diversity of nematodes, protozoans, trematodes, and cestodes, including unidentified strongyles (73%), *Oesophagostomum* spp. (53%), *Ancylostoma* spp. (27%), *Trichuris* spp. (13%), *Ascaris* spp. (13%), *Mammomonogamus* spp. (13%), *Strongyloides* spp. (47%), *Balantidium coli* (20%), *Entamoeba coli* (20%), *Endolimax nana* (6%), *Fasciola hepatica* (6%), *Paramphistomum* spp. (13%), and *Taenia* spp. (6%). Overall, nematodes (six taxa) were more common than protozoa (three taxa), trematodes (two taxa), and cestodes (one taxon).

**Figure-2:**
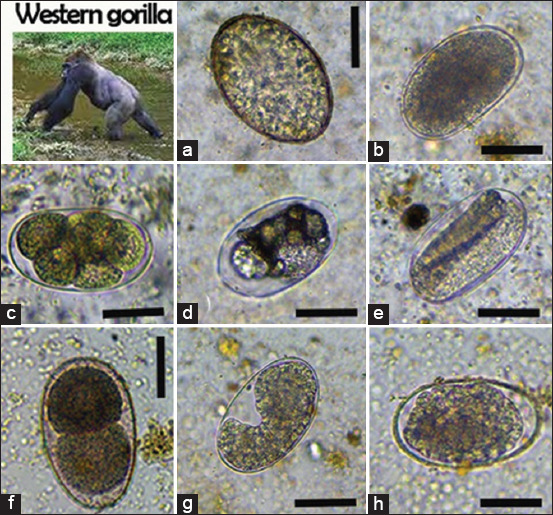
Overview of gastrointestinal parasites found by microscopy in western lowland gorillas: (a) *Paramphistomum* spp., (b) *Oesophagostomum* spp., (c) *Ancylostoma* spp., (d and g) strongylid egg, (e) *Strongyloides* spp., (f) *Mammomonogamus* spp., (h) *Ascaris* spp., Scale bars: 50 μm (a) and 25 μm (b-h).

**Figure-3:**
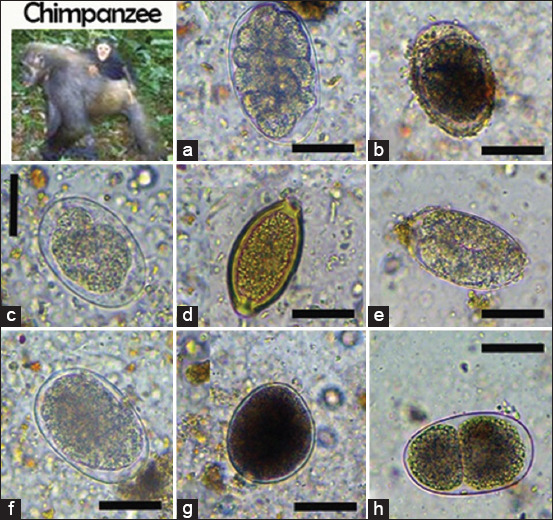
Overview of gastrointestinal parasites found by microscopy in chimpanzees: (a) *Oesophagostomum* spp., (b) *Ascaris* spp., (c and f) strongylid egg, (d) *Trichuris* spp., (e) *Strongyloides* spp., (g) *Balantidium coli*; (h) *Mammomonogamus* spp. Scale bars: 25 μm (a-g).

**Figure-4:**
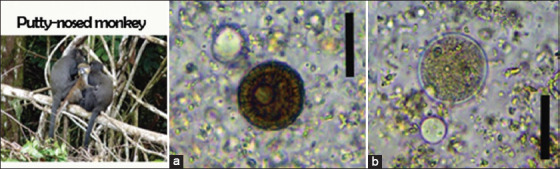
Overview of gastrointestinal parasites found by microscopy in putty-nosed monkeys: (a) *Taenia* spp., (b) *Balantidium coli*, *Entamoeba coli*. Scale bars: 20 μm (a and b).

**Figure-5:**
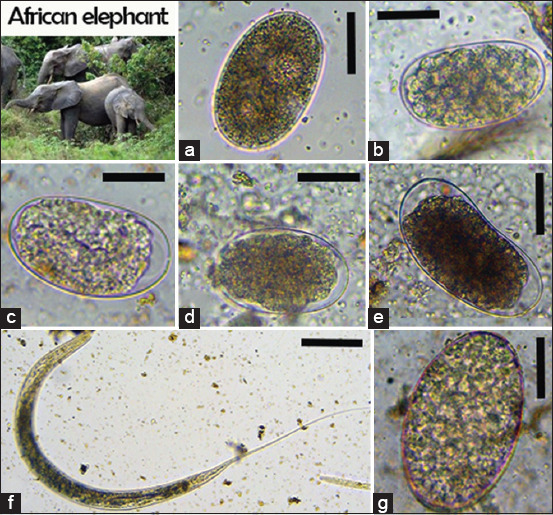
Overview of gastrointestinal parasites found by microscopy in elephants: (a,e) Strongylid egg, (b, d) *Oesophagostomum* spp., (c) *Strongyloides* spp., (f) *Oesophagostomum* larvae; (g) *Paramphistomum* spp. Scale bars: 25 μm (a–e) and 50 μm (g).

**Figure-6:**
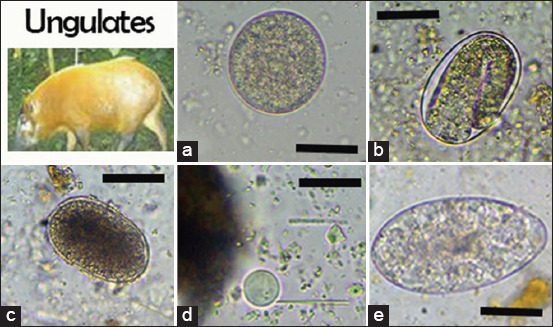
Overview of gastrointestinal parasites found by microscopy in ungulates: (a) *Balantidium coli*, (b) *Strongyloides* spp., (c) *Oesophagostomum* spp., (d) *Entamoeba coli*; (e) *Fasciola hepatica*. Scale bars: 25 μm (a-c), 10 μm (d), and 50 μm (e).

**Figure-7:**
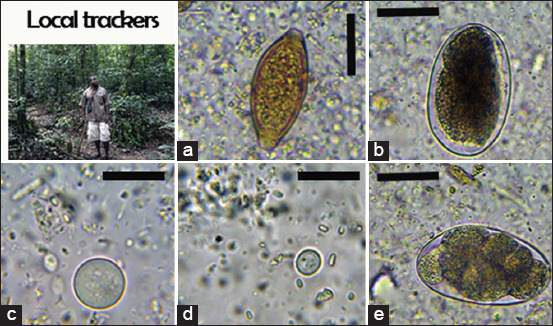
Overview of gastrointestinal parasites found by microscopy in local human trackers: (a) *Trichuris* spp.; (b) *Oesophagostomum* spp.; (c) *Entamoeba coli*; (d) *Endolimax nana*; (e) *Ancylostoma* spp. Scale bars: 25 μm (a-b, e), 15 μm (c), and 10 μm (g-i).

We identified six nematodes (*Trichuris* spp., *Ancylostoma* spp., *Oesophagostomum* spp., *Strongyloides* spp., *Mammomonogamus* spp., and *Ascaris* spp.), two protozoans (*B. coli* and *E. coli*), one trematode (*Paramphistomum* spp.), and one cestode taxon (*Taenia* spp.) in non-human primates (Figures-2-4). In African forest elephants, we identified three nematodes (*Strongyloides* egg, *Oesophagostomum* spp., and *Strongyloides* spp.) and one trematode (*Paramphistomum* spp.) (Figure-5). In ungulates, we identified two nematodes (*Strongyloides* spp. and *Oesophagostomum* spp.), two protozoans (*B. coli* and *E. coli*), and one trematode (*F. hepatica*) (Figure-6). In people working as trackers, we identified three nematodes (*Trichuris* spp., *Oesophagostomum* spp., and *Ancylostoma* spp.) and one protozoan (*E. nana*) (Figure-7).

## Discussion

To the best of our knowledge, this is the first study to assess gastrointestinal parasites in a wide range of mammals living in MDNP, including non-human primates (western gorilla, chimpanzee, and putty-nosed monkey), African forest elephants, ungulates (African buffalo, blue duiker, bay duiker, and red river hog), and people working as trackers. The overall prevalence of parasitic gastrointestinal infection was 60%. This is slightly higher than in the previous studies performed in the same habitat [17,19]. The taxonomic richness of parasites was highest in western gorillas and chimpanzees, followed by elephants and people working as trackers. These differences may result from differences in sampling frequency between the hosts or from other factors that contribute to the development, survival, and dispersal of the infective stages of parasites in the environment.

### Parasites detected

We detected 12 species of gastrointestinal parasites (three protozoans and nine helminths) in the host species studied, of which *Oesophagostomum* spp. was the most prevalent (53%). The detection of infections caused by this parasite is crucial because of its severity and zoonotic potential. Moreover, this nodular worm can cause serious illnesses, resulting in the deaths of non-humans [24-26]. The higher prevalence of *Oesophagostomum* spp. compared with the other parasites that we identified may indicate the greater potential of this parasite to infect a wide range of mammalian species and suggests that it is highly infective and efficiently transmitted.

The second most prevalent intestinal nematode was *Strongyloides* spp., with a prevalence of 47%. This zoonotically significant threadworm is of public health interest because it may lead to severe gastroenteritis, hepatitis, pneumonia, myocarditis, and death [24]. In addition, the mortality rate from such an infection and its comorbidities is >80% [27].

The third most prevalent intestinal nematode was *Ancylostoma* spp. This zoonotic nematode is a blood-feeding parasite that may cause eosinophilic enteritis, and it has been associated with mild-to-severe clinical pathologies, including anemia and diarrhea, protein malnutrition, dysentery, weight loss, and death in primates [28,29].

*B. coli* is a zoonotic parasite that causes severe pathology in the intestinal tract, such as diarrhea, rectal prolapse, and hemorrhagic dysentery, in both humans and animals [30]. *E. coli* is a protozoa that can lead to the death of the host animal in heavy infestations [31].

Other helminth species (*Trichuris* spp., *Mammomonogamus* spp., *Ascaris* spp., and *Paramphistomum* spp.) had a prevalence of 13%, respectively. These parasites are also pathogenic. For example, *Trichuris* spp. was reported to significantly alter the behavior of primate hosts [32], cause intestinal disorder, and even induce death [33]. The clinical manifestations of infections of *Mammomonogamus* spp. include a persistent dry cough accompanied by hemoptysis and asthma, weight loss, pleuritic pain, or nausea [34]. *Ascaris* spp. are one of the most prevalent helminth infections in humans and animals, and infections are associated with diarrhea, malnutrition, and impaired growth and development, which can lead to death [35]. *Paramphistomum* spp. is a trematode that can cause serious clinical disease, especially in heavy infestations [36].

*E. nana*, *Taenia* spp., and *F. hepatica* all had a prevalence of 6%, respectively. *E. nana* is a non-pathogenic ameba. *Taenia* spp. are cestodes associated with the most neglected tropical disease in humans worldwide [37]. *F. hepatica* is associated with intestinal tissue damage, hemorrhage, and death in free-ranging African elephants [36].

### Host species sampled

Our results of intestinal parasite prevalence for western gorillas are similar to those of the previous studies [19]. Little is known about the prevalence of gastrointestinal parasites that infect other mammalian species in the same habitat. However, the parasitic taxa that we identified in non-human primates are all species that can infect other non-human primates and have a high potential for transmission to humans because of their simple life cycles [38,39]. Other than the absence of protozoa in gorillas and chimpanzees, the gastrointestinal nematodes identified in our study are similar to those in the previous studies of the same or closely related populations and cause asymptomatic or mild disorders [19,38,40]. Some of the nematodes that we identified, such as *Strongyloides* spp., *Oesophagostomum* spp., and *Trichuris* spp., commonly parasitize wild apes [38,41]. For instance, *Oesophagostomum* spp. are associated with morbidity and mortality in some primate populations [25].

Our parasitological examinations of African forest elephants found one trematode genus (*Paramphistomum* spp.), two nematode genera (*Oesophagostomum* spp. and *Strongyloides* spp.), and unidentified nematode eggs. Elephants are most commonly parasitized by a diverse range of nematode (*Murshidia* spp., *Mammomonogamus* spp., and *Quilonia* spp.) and trematodes (*Protofasciola* spp., *Brumptia* spp.) [42]. Despite the absence of these parasites in the African forest elephants in our study, our findings correlate with several previous reports of gastrointestinal parasites in African elephants, where the prevalence of nematode infections was 2-3 times greater than trematode infections [42,43]. Moreover, the absence of these nematodes and trematodes may be due to several mechanisms that depend on environmental conditions [42] or be attributed to our small sample size.

In ungulates, we identified two nematode genera that have been frequently reported (*Strongyloides* spp. and *Oesophagostomum* spp.) and two protozoa genera that have been documented less often in this group (*B. coli* and *E. coli*). These results correlate with studies showing that ungulates are infested with a wide variety of gastrointestinal parasites [44]. Nevertheless, we did not identify gastrointestinal nematodes that typically parasitize several species of ungulates, such as *Moniezia* spp., *Cooperia* spp., and *Teladorsagia* spp. This lack of detection may be due to our small sample size.

The fecal analysis of local trackers working in and around MDNP revealed the presence of three nematode genera (*Trichuris* spp., *Oesophagostomum* spp., and *Ancylostoma* spp.) and one protozoan (*E. nana*). Nematodes were prevalent in all of the sampled mammal species. The presence of these nematodes in both non-human primates and humans suggests the occurrence of multiple cryptic species that may infect both humans and non-human primates. This supports the hypothesis that indirect contact between humans and animals sharing habitats through frequent tourist visits and assistants and researchers conducting field studies increase the possibility of infection by the same parasites [45,46].

## Conclusion

Our results show that mammalian species in MDNP are exposed to and infected by a diverse range of gastrointestinal parasites that may be reciprocally transmitted between species. Nevertheless, because of the low sample size (a total of 25 samples from eight host species), our results should be cautiously interpreted. In the future, we plan to perform a comprehensive longitudinal study of parasites in MDNP because of their importance to conservation and ultimately to the survival of wild mammals. Finally, molecular studies are necessary to confidently distinguish the species of gastrointestinal parasites and to test the hypothesis that cross-infection occurs in this area.

## Authors’ Contributions

SED, UM, TT, and BN: Conceived and designed the study as well as collected the samples. SED, CM, and LB: Carried out the parasitological analysis. SED and DB: Carried out the morphological identification analysis. SED and UM: Supervised, reviewed, and edited the manuscript. SN, BN, ROZ, and DK: Prepared funding acquisition and project administration. All authors read and approved the final manuscript.
